# Role of lupus retinopathy in systemic lupus erythematosus

**DOI:** 10.1186/s12348-016-0081-4

**Published:** 2016-05-12

**Authors:** Ranju Kharel (Sitaula), Dev Narayan Shah, Divya Singh

**Affiliations:** Department of Ophthalmology, B. P. Koirala Lions Centre for Ophthalmic Studies, Tribhuvan University Institute of Medicine, PO Box-5889, Maharajgunj, Kathmandu Nepal; Department of Internal Medicine, Tribhuvan University Teaching Hospital, Institute of Medicine, Maharajgunj, Kathmandu Nepal

**Keywords:** Autoimmune, Fundus, Lupus retinopathy, Renal, Systemic lupus erythematosus

## Abstract

**Background:**

Lupus retinopathy is one of the most common vision-threatening complications of systemic lupus erythematosus. The presence of lupus retinopathy is an accurate guide to the presence of active systemic disease activity.

**Results:**

A prospective study was conducted looking at 91 established cases of systemic lupus erythematosus to evaluate lupus retinopathy. The patients were divided into two groups according to the presence or absence of lupus retinopathy, and a comparison of clinical and laboratory findings between two groups was done. Among 91 SLE patients, 5 were male and 86 were female; of which, 85 (93.4 %) were outpatients and 6 (6.6 %) were inpatients. Lupus retinopathy was found in 13 eyes of 11 cases out of 91 cases (12.1 %). Among these 13 eyes with lupus retinopathy, 61.5 % had mild type of lupus retinopathy, 15.4 % had moderate type, and 23.1 % had severe lupus retinopathy.

The mean age of the cases at ophthalmological examination with and without retinopathy was 30.4 and 31.9 years, respectively. The mean serum creatinine level was 190.4 μmol/l which was higher than in the patients without retinopathy (96.2 μmol/l). The mean ESR in patients with retinopathy was higher than without retinopathy (34.2 vs. 32). Similarly, the mean platelet count in SLE patients with retinopathy was 154,245/μl and in SLE patients without retinopathy was 135,828/μl.

**Conclusions:**

Retinal lesions in SLE patients are of critical importance, both visually and prognostically.

## Background

Systemic lupus erythematosus (SLE) is a chronic, autoimmune, connective tissue disorder affecting multiple organ systems often with a relapsing and remitting clinical course. SLE is often called a “woman’s disease” because 90 % of affected individuals are females [1]. Manifestations of SLE are protean and any organ or system of the body including the eye can be affected due to the inflammatory response to the circulating immune complexes [1].

Ocular manifestations are a marker for overall systemic disease activity and can occur in up 1/3rd of all SLE patients. The incidence of retinal involvement in SLE is 7–26 % [2] and is the second most common ocular manifestation after keratoconjunctivitis sicca [3]. It is one of the most common vision-threatening complications of systemic lupus erythematosus with an incidence of up to 29 % in patients with active systemic disease [3]. The presence of active SLE retinal vasculopathy is an extremely accurate guide to the presence of systemic disease activity, occult or overt. Additionally, life-table survival estimates have shown decreased survival in patients with SLE retinopathy, compared to SLE patients without retinopathy [3]. Retinal lesions in SLE patients, therefore, are of critical importance, both visually and prognostically [4].

The control of systemic SLE is the mainstay of treatment for lupus retinopathy which can be achieved by systemic corticosteroids and immunosuppression [3]. Initial treatment is usually with oral corticosteroids (prednisolone 1 mg/kg/day), but may be preceded by intravenous methylprednisolone (500 mg–1 g daily for 3 days). This is then supplemented with, or replaced by, other immunosuppressive agents as part of a steroid-sparing strategy or for resistant disease [3]. Periocular steroid injections may have a role in unilateral/asymmetric disease; however, they should be used cautiously and avoided in patients with necrotising scleritis [2]. In the presence of significant vaso-occlusive disease with the presence of antiphospholipid antibody, anti-coagulation and the addition of low-dose acetylsalicylic acid may be beneficial [5]. Proliferative retinopathy usually requires treatment with laser photocoagulation akin to the treatment of proliferative diabetic retinopathy [6].

## Methods

A prospective study was conducted among the established cases of SLE referred to the eye department from the various departments of Tribhuvan University Teaching Hospital between 2008 and 2010. After dilated fundus evaluation, presence or absence of lupus retinopathy was identified and grading was done. Mild lupus retinopathy consists of cotton–wool spots, perivascular hard exudates, retinal hemorrhages, and vascular tortuosity [7]. Moderate lupus retinopathy has focal or generalized arteriolar constriction and venous tortuosity. At the severe end of the spectrum, there is occlusion of retinal arterioles and consequent retinal infarction, termed vaso-occlusive retinopathy [8] causing artery/vein occlusions, vitreous hemorrhage, retinal ischemia, and neovascularisation [8–11].

The patients were divided into two groups according to the presence or absence of lupus retinopathy, and a comparison of clinical and laboratory findings between two groups was done. Patients were followed up for 2 years.

### Patients

Ninety-one consecutive patients with SLE (5 male, 86 female) who fulfilled the American College of Rheumatology criteria for SLE [11] were enrolled; 85 outpatients (93.4 %) and six were inpatients (6.6 %). The mean age of female and male cases at the ophthalmological examinations were 26.7 (SD ± 10.3) years and 24.4 (SD ± 12.1) years, respectively.

Thirty-one patients (34.1 %) were receiving corticosteroids; 30 patients (32.96 %) were treated with combination of corticosteroid with antimalarial (14.3 %) and/or antihypertensive agents (12.1 %). Intravenous methylprednisolone was administered in four cases (4.4 %). The mean duration of steroid treatment was 2.1 years. The daily dose of oral corticosteroid at the ophthalmological examination ranged between 10 and 40 mg.

Nine patients (9.9 %) were under immunosuppressant drugs, including cyclophosphamide and azathioprine; six patients (6.6 %) with CNS involvement were under antiepileptics. However, 20 patients (21.9 %) were without any medication as they were referred to the eye department to rule out any contraindication before starting hydroxychloroquine.

### Statistical analysis

The data were analyzed with SPSS 20 version, and *t* test and Fisher’s 2 × 2 test were used to analyze *p* value.

## Results

A total of 91 SLE patients (182 eyes) were examined to evaluate the presence of lupus retinopathy.

### Incidence and features of lupus retinopathy

Lupus retinopathy was found in 11/91 (12.1 %) patients with female to male ratio of 10:1. The mean age of the cases at ophthalmological examination with and without retinopathy was 30.4 and 31.9 years, respectively.

Among them, eight cases (72.7 %) were examined in outpatients settings and three (27.3 %) were indoor cases (Table 1).

**Table 1:** Backgrounds of the patients with systemic lupus erythematosus (SLE)

	Lupus retinopathy	
	Absent	Present
Number of SLE patients	80	11
Number of SLE admitted cases	6/91 (6.6 %)	3/11 (27.3 %)
Age at onset of SLE (years)	31.9	30.4
Oral Steroid dose at ophthalmological examination (range)	5–50 mg	10–40 mg
History of intravenous steroid	3/91 (3.3 %)	1/11 (9.1 %)
History of immunosuppressant treatment	8/91 (8.8 %)	1/11 (9.1 %)

Among the cases with retinopathy, nine patients had unilateral retinopathy and two patients had bilateral lupus retinopathy. The mean IOP was normal.

### Retinal findings of the patients with lupus retinopathy (Table 2)

Among these 13 eyes of 11 patients of lupus retinopathy, commonest was the mild type in eight eyes (61.5 %) followed by moderate type in two eyes (15.4 %) and severe type in three eyes (23.1 %). Among severe lupus retinopathy, two eyes had retinal vasculitis and one eye had central retinal arterial occlusion (CRAO).

**Table 2:** Backgrounds of the retinal findings in SLE

Retinal findings in SLE					
	Mild lupus retinopathy		Moderate lupus retinopathy	Severe lupus retinopathy	
Patient no.	Cotton–wool spot	Hard exudates	Arteriolar attenuation	Vasculitis	Arterial occlusion
1	+	+			
2	+	+			
3				+	
4				+	
5					+
6	+	+			
7^a^	+	+	+		
8	+	+			
9	+	+			
10^a^	+	+	+		
11	+	+			

^a^In two patients, one eye had cotton–wool spot with hard exudates and the other eye had arteriolar attenuation

### Systems affected in SLE retinopathy (Table 3)

In our 91 SLE patients, renal involvement was found in 59.3 % cases and central nervous system in 11 %. But in those 11 cases with lupus retinopathy, 100 % renal and 27.3 % central nervous system involvement was found. So, all our cases of lupus retinopathy had renal dysfunction in the form of protenuria or a raised creatinine level. The CNS findings observed in lupus retinopathy cases were chorea, convulsion, and epilepsy.

**Table 3:** Systemic involvement in lupus retinopathy

Patient no.	Age/sex	Lupus retinopathy	Renal involvement	CNS involvement
1	F/54	LE mild retinopathy	+	–
2	F/24	RE mild retinopathy	+	–
3	F/40	RE severe retinopathy	+	–
4	F/42	LE severe retinopathy	+	–
5	F/27	BE mild retinopathy	+	+
6	F/44	RE mild retinopathy	+	−
7	F/30	RE mild and LE moderate retinopathy	+	+
8	M/13	RE mild retinopathy	+	−
9	F/22	RE mild retinopathy	+	−
10	F/24	RE moderate and LE mild retinopathy	+	+
11	F/26	RE mild retinopathy	+	−

The mean serum creatinine level was 190.4 μmol/l which was higher than in the patients without retinopathy (96.2 μmol/l). The mean ESR in patients with retinopathy was higher than without retinopathy (34.2 vs. 32). Similarly, the mean platelet count in SLE patients with retinopathy was 154,245/μl and in SLE patients without retinopathy was 135,828/μl (Table 4).

**Table 4:** Comparison of significance test in SLE with or without retinopathy

	SLE without retinopathy	SLE with retinopathy	Remarks on *p* value
Renal system involvement	54/91 (59.3 %)	11/11 (100 %)	0.003 (*p* value statistically significant)
CNS involvement	10/91 (11 %)	3/11 (27.3 %)	.347
Mean ESR	32.03	34.18	.660
Mean platelet count (per μl)	135,828	154,245	.649
Mean serum creatinine (μmol/l)	96.2 μmol/l	190.4 μmol/l	.001 (*p* value statistically significant)

### Association with autoantibodies

SLE is generally considered to be an immune complex-mediated disease where the lupus retinopathy occurs due to immune complex deposition in retinal vessels in SLE [12]. The incidence of antinuclear antibody (ANA) and anti-dsDNA antibody in SLE patients with retinal vascular disease versus without retinal vascular disease was 87.5 and 72.7 %. The higher positive antiphospholipid antibodies were seen in SLE patients in those with retinopathy compared to those without retinopathy was 18.1 versus 4.4 %. But all of them were not statistically significant.

## Discussion

Retinopathy is one of the important manifestations of SLE, which develops with an incidence of 7–26 % [4] of SLE patients, although the frequency of the findings varies depending on the patient population being studied and systemic disease activity [12]. According to Ushiyama et al. [13], the incidence of lupus retinopathy is 10 % (7/69) which is similar to our incidence of lupus retinopathy {12.1 % (11/91)}. Our study reveals incidence of lupus retinopathy in admitted patients to be 27.3 % which correlated with the other literature where lupus retinopathy in the admitted patients could be as high as 29 % [14].

Ushiyama et al. [13] reported the mean age at onset of SLE in the patients with and without retinopathy to be 34.2 and 31.9 years, respectively, and all of them were females. This is comparable with our study where the mean ages at onset of SLE in our patients were 30.4 and 31.9 years, respectively, of them, 10 were females and 1 male. Sex hormones are known to affect the clinical course of SLE; estrogen enhances the disease and testosterone suppresses it [15, 16] and 90 % of women are of child-bearing age [17].

Retinopathy in SLE is suggestive of high disease activity during the course of SLE, and hence, is a marker of poor prognosis for survival, that is SLE patients with retinopathy have overall worse prognosis and decreased survival, compared to SLE patients without retinopathy [3]. Interestingly, the severe form of vaso-occlusive retinopathy is associated with similar changes in the central nervous system vasculature [8, 18, 19].

We found that the renal dysfunction in SLE without retinopathy was 59.3 %, but in patients with retinopathy was 100 %. Although it is unclear whether lupus retinopathy is directly associated with renal disease, retinopathy may develop in severe SLE which also involves the kidney or CNS, or both. It has a major impact on morbidity and mortality.

Several studies report that retinal vascular disease in SLE patient parallels systemic disease activity [14, 20] and that the effective treatment of the systemic disease, along with simultaneous control of any associated systemic hypertension, results also in concurrent decrease in the retinal lesions, especially the disappearance of cotton–wool spots [9, 14].

The serologic hallmark of SLE is the presence of ANAs in highly sensitive and useful screening tool. Anti-dsDNA antibody is SLE specific and correlates with disease activity [21]. The antiphospholipid antibody is thrombogenic and is associated with both CNS and ocular vaso-occlusive phenomena [18] that can manifest as visual disturbances, transient monocular blindness, and chorioretinopathy in patients with SLE [22]. A higher incidence of antiphospholipid antibodies in SLE patients with retinal vascular disease, compared to those without (77 vs. 29 %), has been reported [23] and in our study also the incidence was 18.1 versus 4.4 %.

## Conclusions

The ocular fundus is the only part of the human body where we can directly observe small vessels without injuring the tissue. And lupus retinopathy reflects systemic vascular damage, so ocular fundus examination should be regularly done. Finally, the eyes may be the harbinger of a potentially severe systemic flare-up of SLE, so careful monitoring is needed. A symbiotic relationship among rheumatologist, physicians, dermatologist, and ophthalmologist may be formed to better understand the disease status and plan the treatment strategy of the patients with SLE.
